# Obsessed with Healthy Eating: A Systematic Review of Observational Studies Assessing Orthorexia Nervosa in Patients with Diabetes Mellitus

**DOI:** 10.3390/nu13113823

**Published:** 2021-10-27

**Authors:** Maria G. Grammatikopoulou, Konstantinos Gkiouras, Georgia Polychronidou, Chrysi Kaparounaki, Kalliopi K. Gkouskou, Faidon Magkos, Lorenzo Maria Donini, Aristides G. Eliopoulos, Dimitrios G. Goulis

**Affiliations:** 1 Department of Nutritional Sciences & Dietetics, Faculty of Health Sciences, Alexander Campus, International Hellenic University, GR-57400 Thessaloniki, Greece; mariagram@auth.gr; 2 Unit of Reproductive Endocrinology, 1st Department of Obstetrics and Gynecology, Medical School, Aristotle University of Thessaloniki, Papageorgiou General Hospital, GR-56429 Thessaloniki, Greece; 3 Medical School, Faculty of Health Sciences, University Campus, Aristotle University of Thessaloniki, GR-54124 Thessaloniki, Greece; kostasgkiouras@hotmail.com (K.G.); polipoligeorgia@gmail.com (G.P.); chryssa.kap@gmail.com (C.K.); 4 Laboratory of Biology, School of Medicine, National and Kapodistrian University of Athens, GR-11527 Athens, Greece; gkouskoukal@med.uoa.gr (K.K.G.); eliopag@med.uoa.gr (A.G.E.); 5 Embiodiagnostics Biology Research Company, GR-71305 Heraklion, Greece; 6 Department of Nutrition, Exercise and Sports, Faculty of Science, University of Copenhagen, 1958 Frederiksberg, Denmark; fma@nexs.ku.dk; 7 Department of Experimental Medicine-Medical Pathophysiology, Food Science and Endocrinology Section, Sapienza University of Rome, 00185 Rome, Italy; lorenzomaria.donini@uniroma1.it; 8 Biomedical Research Foundation of the Academy of Athens, GR-11527 Athens, Greece; 9 Center for New Biotechnologies and Precision Medicine, School of Medicine, National and Kapodistrian University of Athens, GR-11527 Athens, Greece

**Keywords:** disordered eating, eating disorders, metabolic control, healthism, psychology, eating behavior, anorexia nervosa, binge eating disorder, bulimia nervosa, diet, purging

## Abstract

Orthorexia nervosa (ON) is an unspecified feeding or eating disorder (USFED) characterized by an exaggerated, unhealthy obsession with healthy eating. Τypical eating disorders (EDs) and USFEDs are common among patients with diabetes mellitus (DM), which complicates metabolic control and disease outcomes. The present systematic review summarizes the evidence on the prevalence of ON symptomatology among patients with DM. PubMed, Web of Science, Scopus, and grey literature were searched, and relevant observational studies were screened using the Rayyan software. The quality of the studies was assessed using the appraisal tool for cross-sectional studies (AXIS) and the Newcastle–Ottawa scale (NOS). Out of 4642 studies, 6 fulfilled the predefined criteria and were included in the qualitative synthesis. Most studies relied on the ORTO-15 or its adaptations to identify ON among patients with DM. No apparent sex or age differences exist regarding the prevalence of ON symptoms. None of the studies compared the prevalence of ON in patients with type 1 and type 2 DM. Most of the research was of average to good methodological quality. In conclusion, patients with DM often exhibit ON tendencies, although research is still limited regarding the etiology or mechanistic drivers behind ON and the characteristics of patients with a dual ON–DM diagnosis.

## 1. Introduction

Orthorexia nervosa (ON) is an atypical eating disorder (ED) that belongs to the group of unspecified feeding and eating disorders (USFED) [1]. It was first described by Bratman [2] as an exaggerated, unhealthy obsession with healthy eating. The difference between adhering to healthy eating principles versus ON is that, in the latter case, the affected individual might be driven by dietary asceticism, cherry-picked evidence, or even by evidence-based recommendations, leading to a restrictive dietary pattern in pursuit of improved health [3]. Moreover, ON often has an underlying psychopathology, with a frequent overlap of symptoms between ON and anorexia nervosa (AN), obsessive–compulsive disorder (OCD), somatic symptom disorder, illness anxiety disorder, and psychotic spectrum disorders [4], leading to the development of ON as a manifestation of “healthism” [5].

On the other hand, healthy eating comprises the first-line prevention for various non-communicable diseases, including diabetes mellitus (DM). Adherence to a healthy diet is an integral part of the effective self-management for patients with prediabetes, type 1 diabetes mellitus (T1DM), type 2 diabetes mellitus (T2DM), and gestational diabetes mellitus (GDM) [6,7,8]. Due to the need for the frequent monitoring of blood glucose concentrations before and after each meal and the required adherence to a healthy diet regime, patients with T1DM often report feeling excessively preoccupied with their diet [9,10]. Moreover, in T2DM in particular, disordered eating patterns are quite common, and span a wider range of symptoms than those of patients with T1DM. Individuals with T2DM are more likely to report poorer self-efficacy for following the dietary recommendations set by experts, instead alternating between binge-eating disorder (BED) and night-eating syndrome [11,12].

Overall, it appears that EDs often coincide with DM, leading to “corrective” practices such as the use of laxatives or diuretics, bingeing, vomiting [13], engaging in excessive exercise [14], and even withholding insulin [15]; this is referred to as diabulimia [16,17,18]. Moreover, in a population of patients with T1DM and EDs, 93.8% reported being diagnosed with DM before their ED diagnosis, suggesting a increasing psychopathology as a possible epiphenomenon of DM diagnoses among ED-prone individuals [14,19].

According to a Danish and Swedish cohort study of more than 4,300,000 individuals [20], patients with T1DM exhibited a greater risk of having an ED diagnosis. Similar findings have also been confirmed in other populations [21,22,23,24]. Distinct forms of EDs, including bulimia nervosa (BN), BED and AN tend to aggregate in families, with twin studies indicating that 40–60% of the prevalence of EDs is associated with heritability [25]. Although these forms of EDs share patterns of psychiatric/behavioral and anthropometric characteristics and are frequently assimilated, their biological underpinnings are likely to differ [26]. It appears that when clusters of autoimmune diseases are apparent, a patient’s risk of exhibiting disordered eating behaviors is further increased compared to that of being diagnosed with T1DM alone [23]. However, it was not until recently that analyses of large-scale genetic and phenotypic data pointed to shared pathophysiological mechanisms for DM and disordered eating. A meta-analysis of 12 cohorts (a total of 3495 AN cases and 10,982 controls) identified one locus on chromosome 12 (SNP rs4622308, FAM19A2) that has previously been associated with T1DM [27]. Other risk loci were associated with psychiatric disorders, physical activity, and metabolic (including glycemic) traits, which have led to a reconceptualization of AN as a metabolo-psychiatric disorder [28]. Thus, it appears that, beyond the triggering of disordered eating constituting an epiphenomenon of disease-related stress, genetic predisposition also links DM with EDs.

Since the co-existence of DM with EDs (typical or atypical) appears to be quite common, the present systematic review aimed to summarize the literature on the prevalence and symptomatology of ON in patients with a DM diagnosis. The research question was, “What is the prevalence of ON in patients with DM, and what are the associated conditions/signs in this population?”

## 2. Materials and Methods

### 2.1. Systematic Review Protocol and PIO

The Preferred Reporting Items for Systematic reviews and Meta-Analyses (PRISMA) was used for the present review. The study’s protocol was published on the Open Science Framework (OSF) website (https://osf.io/p8mu9/, accessed on 2 October 2021).

The PIO describing the study’s research question is detailed in Table 1.

### 2.2. Search Strategy

Studies related to the research question were identified through searches in PubMed, Web of Science, Scopus, and the grey literature (including conference proceedings, Endocrine Abstracts, theses, etc.), from searches from the study’s inception until July 2021, by two independent reviewers (G.P. and C.K.). In September 2021, a confirmatory search was conducted in order to include possible new studies. Any disagreement between reviewers was resolved by two senior researchers (K.G. and M.G.G.).

Rayyan [29], a web and mobile app for systematic reviews, scanned and identified studies fulfilling the study’s criteria. The cited references identified were imported to Rayyan, and duplicate entries were removed.

Search terms and keywords were derived using the SPIDER (Sample, Phenomenon of Interest, Design, Evaluation, Research type) framework [30] (Table 2).

**Figure 1:**
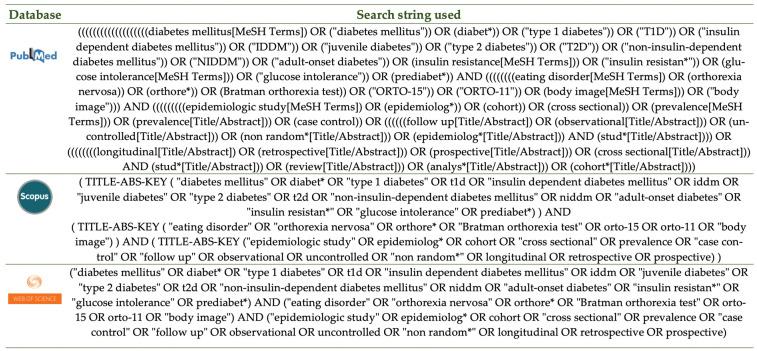
The search strategy applied in the three databases.

The applied keywords were either relevant to the research question—including “diabetes mellitus”, “type 1 diabetes”, “insulin-dependent diabetes mellitus”, “juvenile diabetes”, “type 2 diabetes”, “non-insulin-dependent diabetes mellitus”, “adult-onset diabetes”, “insulin resistance”, “glucose intolerance”, “prediabetes”, “orthorexia nervosa”, “eating disorder”, “Bratman orthorexia test”, “ORTO-15”, “ORTO-11”, and “body image”—or relevant to the observational study types used—including “epidemiologic study”, “cohort”, “cross-sectional”, “case–control”, “prevalence”, “observational”, “follow up”, “longitudinal”, “retrospective”, “prospective”, “uncontrolled”, “non-random”, “study”, “review”, and “analysis”. Wherever applicable, MeSH terms and abbreviations were also used. Figure 1 details the search string used in each database.

### 2.3. Inclusion and Exclusion Criteria

Studies were included in the synthesis when they: (1) used a population of patients with a prediabetes or DM (T1DM/T2DM) diagnosis, (2) included patients irrespective of their age; (3) evaluated orthorexic tendencies using any tool (due to a lack of a consensus on diagnostic criteria); (4) used a cross-sectional research design (for the main and secondary outcomes) or a cohort–/case–control design (for the secondary outcomes); (5) were published in any language; (6) were in either abstract or full-text format, (7) were published before September 2021.

The criteria for excluding studies were: (1) they evaluated eating disorders (typical or atypical, including other specified feeding or eating disorders [OSFED] or USFED) other than ON; (2) they used other research designs (randomized clinical trials, time-series) or reviews; and (3) they used samples of patients with a different diabetes diagnosis (e.g., GDM).

### 2.4. Quality Assessment of the Studies

The methodological quality of the included studies was assessed by two independent reviewers using the critical appraisal tool for assessing the quality of cross-sectional studies (AXIS) [31] and the Newcastle–Ottawa scale (NOS) for assessing case–control studies [32].

### 2.5. Data Extraction

Two researchers independently extracted data in predefined excel spreadsheets. Information regarding the sample (size, diabetes type, age, and % female); recruitment (site, time period); country of origin; DM therapy (insulin/diet/medication); tools used to evaluate ON tendencies, prevalence, or score of ON tendencies; and general results associated with ON, were extracted for all studies.

### 2.6. Data Synthesis

No minimum or maximum sample size requirement was imposed. The primary outcome variable was the prevalence of ON in the included cross-sectional studies. If a meta-analysis was feasible, the ON prevalence would be presented as event rates.

## 3. Results

### 3.1. Search Results

**Figure 2:**
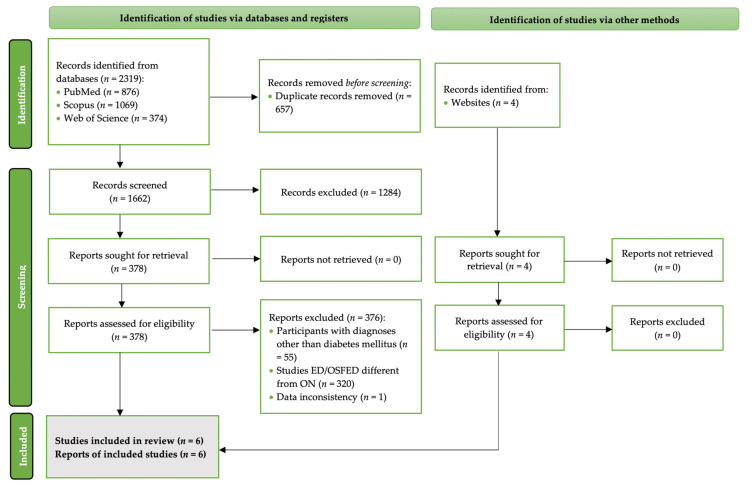
The PRISMA 2020 flowchart of the study selection process [33].

Out of 4642 studies in total, 6 fulfilled the criteria and were included in the present review. Figure 2 details the PRISMA 2020 flow diagram of the study selection process [33].

### 3.2. Research on ON among Patients with Prediabetes/DM

None of the studies used a population with prediabetes. Two studies focused on patients with a T1DM diagnosis [34,35], one used a mixed sample of T1DM and T2DM patients [36], one did not report the exactDM type of participants [37], and the remaining studies assessed ON in patients with a T2DM diagnosis [38,39]. 

**Figure 3:**
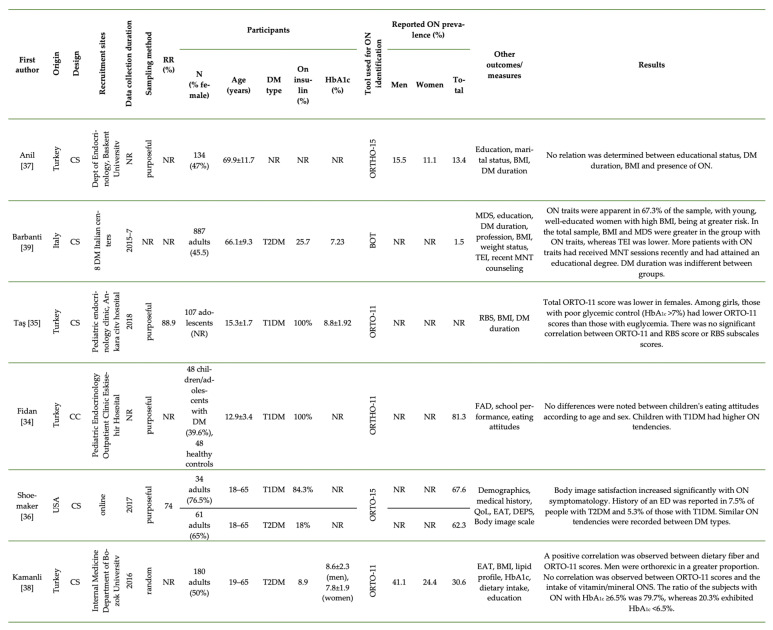
Primary studies assessing ON among patients with DM. BMI, Body Mass Index; BOT, Bratman Orthorexia Scale [2]; CC, case-control; CS, cross-sectional; DEPS, Diabetes Eating Problem Survey; DM, Diabetes Mellitus; EAT, Eating Attitudes Test; ED, eating disorder; FAD, family assessment device; HbA_1c_, glycosylated hemoglobin; MDS, Mediterranean diet score; MNT, medical nutrition therapy; NOD, not other defined; NR, not reported; ON, orthorexia nervosa; ONS, oral nutrient supplements; ORTO, orthorexia questionnaire [40]; QoL, quality of life; RBS, Risk Behavior Scale; T1DM, Type 1 Diabetes Mellitus; T2DM, Type 2 Diabetes Mellitus; TEI, total energy intake.

Figure 3 describes the available primary cross-sectional research on the prevalence of ON in patients with DM. One study was published in poster format [37], three were full-text articles [34,35,39], and two were student theses [36,38]. All of the studies were published in the English language except for one, which was in Turkish [38].

Most of the research had a cross-sectional design, except one [34], which was a case–control study.

The majority of studies were conducted in Turkey [34,35,37,38], but one was USA-based [36] and another originated from Italy [39]. Most of the researches used adult samples [36,37,38,39], except for the studies conducted by Fidan [34] and Taş [35]—both of which used pediatric populations.

### 3.3. Tools Used to Identify ON Tendencies

Despite the plethora of tools used to identify ON [41], most studies relied on the ORTO-15 or its adaptations, as seen in research on the general population [42]. Specifically, one study used the ORTO-15 [36]; two studies used the Turkish adaptation of the ORTO-15 (ORTO-11) [35,38]; and Fidan [34] and Anil [37] used the ORTHO-11 and ORTHO-15, respectively—probably misspelt variations of the ORTO-15 tool. Barbanti [39] was the only study that applied the Bratman Orthorexia Test (BOT) [2]. None of the studies used the more recently developed ORTO-R [43], the Test of Orthorexia Nervosa (TON-17) [44], the Orthorexia Nervosa Inventory (ONI) [45], the Barcelona Orthorexia Scale (BOS) [46], the Düsseldorf Orthorexia Scale (DOS) [47], the Eating Habits Questionnaire (EHQ) [48], or the Teruel Orthorexia Scale [49].

Due to the lack of a consensus concerning the definition of ON, an overview of the signs and symptoms associated with the condition is elusive; as a result, tools used to identify ON tendencies cannot be 100% disease-specific.

### 3.4. Prevalence of ON Tendencies

Except for Taş [35], all studies provided the prevalence rates of ON in their samples, which ranged from as low as 1.5% in adults with T2DM with the use of the BOT [39] to 81.3% in children and adolescents with T1DM using the ORTO-15 [34]. However, given the lack of a specific ON definition, it is risky to rely on these tools to diagnose ON using cutoffs [43,50,51]. Instead, the results of ON tests can be used in a scale form to identify ON tendencies. Along these lines, Taş’s [35] research was the only study using the ORTO-11 in scale form, but it did not report prevalence rates.

### 3.5. Gender Differences

In a sample of patients with DM, Anil [37] demonstrated increased ON tendencies in men compared to women. A similar finding was noted by Kamanli [38] in patients with T2DM. However, using the BOT, Barbanti [39] revealed greater ON tendencies among women than men.

In a sample of youngsters diagnosed with T1DM, Fidan [34] did not identify gender segregation, whereas Taş [35] reported a lower ORTO-11 score among female participants, indicative of increased ON tendencies.

### 3.6. Body Image and Adiposity

Using a combined sample of patients with T1DM and T2DM, Shoemaker [36] suggested that body image satisfaction increased with ON symptomatology. On the other hand, Barbanti [39] reported a positive association between ON and increased BMI among patients with T2DM.

### 3.7. Glycemic Control

Kamanli [38] noted that only a small proportion (20.3%) of adults with ON tendencies and T2DM achieved adequate glycemic control (HbA1c < 6.5%). Similarly, among adolescents with T1DM [35], girls with poor glycemic control (HbA_1c_ > 7%) exhibited lower ORTO-11 scores—indicative of greater ON tendencies—than those with euglycemia.

### 3.8. Effect of Age

Disordered eating is a common problem globally, especially among young women in pursuit of a thinner, more acceptable body ideal. Similarly, young women with a T1DM diagnosis appear to be prone to EDs [15] and OSFED more frequently compared with their male counterparts. Concerning ON however, the majority of studies failed to identify any age differences. On the other hand, Barbanti [39] reported greater ON tendencies among younger patients with T2DM.

### 3.9. Educational Status

Anil [37] failed to associate educational status with ON tendencies among patients with a DM diagnosis in Turkey, although in Italy [39] women with a higher educational attainment demonstrated more ON traits. The effect of educational status was also apparent in another study of patients with T2DM in Turkey, although the exact differences were not defined by the authors [38].

### 3.10. Dietary Intake and Supplement Use

There was no association between eating attitudes and ON [34]. On the other hand, research in patients with T2DM revealed a positive correlation between dietary fiber intake and ORTO-11 scores [38]. Moreover, Barbanti [39] reported that patients with T2DM and ON traits consumed less energy from foods and drinks compared with those not exhibiting ON traits.

While carefully balancing their nutrient intake via supplementation in pursuit of better health would be expected in these individuals, only one study assessing ON tendencies in patients with DM evaluated oral nutrient supplementation (ONS) intake. In this study, researchers failed to relate dietary supplementation to increased ON tendencies among adults with T2DM [38].

### 3.11. Differences between Patients with T1DM and T2DM

None of the studies assessing ON tendencies have compared patients with distinct types of DM; thus, it is difficult to understand differences in the prevalence or attitudes between patients with T1DM and T2DM.

### 3.12. Quality of Studies

**Figure 4:**
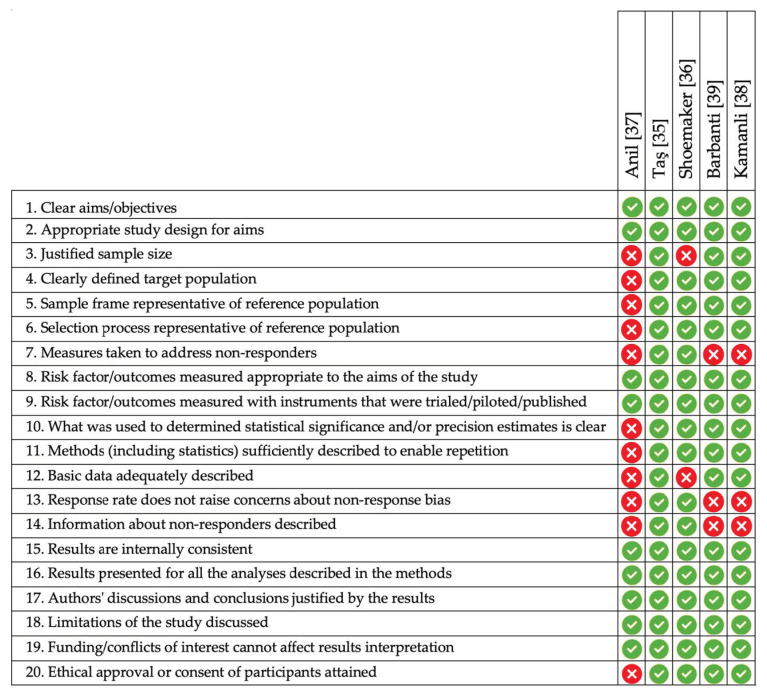
A summary of the quality assessment of the included cross-sectional studies using the AXIS [31] tool.

A summary of the quality assessment of the included cross-sectional studies based on the AXIS is presented in Figure 4. All studies used an appropriate design to answer their research questions. Only one study used a random selection procedure for the participants.

**Figure 5:**
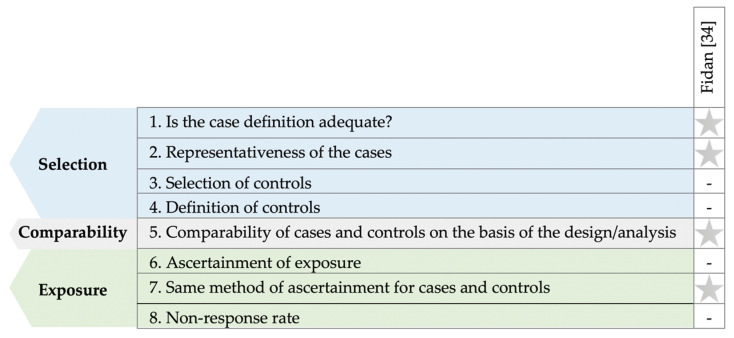
Summary of the quality assessment of included case-control studies using the NOS [32] tool.

Figure 5 details the quality assessment of the included case–control study [34] using the NOS [32]. In half of the domains, the study received one star, with the remainder not being accounted for by the authors.

## 4. Discussion

The current literature indicates that patients with DM may exhibit ON tendencies, although research is still limited regarding the etiology or mechanistic drivers of ON, or the characteristics of patients with a dual diagnosis. The evidence of ON in patients with DM is scanty, as the condition is fairly new and still vies for a distinct diagnosis in the Diagnostic and Statistical Manual for Mental Disorders (DSM-5) [3]. The same is true for conditions associated with the dual DM–ON diagnosis.

ON is characterized by a fixation on food quality, including foods’ nutritional value and perceived “purity” [4]. These features occur irrespective of religious or ecological beliefs and are prompted by an excessive preoccupation with achieving health [4]. Thus, this attitude differentiates patients with DM who adhere to the typical lifestyle guidelines from those obsessed with following a healthy diet. Interestingly, people who self-diagnose themselves with ON have described the condition as a “salvation” from an underlying chronic disease and instead a pursuit of health [52]. According to Pinhas-Hamiel [53], an ED/OSFED/USFED, other specified feeding or eating disorder (OSFED), or UFSED diagnosis is a difficult task for individuals with DM, since disordered eating behaviors are frequently well-hidden and denied.

### 4.1. Characteristics of Patients with DM and ON Tendencies

Although ambiguity is demonstrated in the existing research, the coexistence of EDs/OSFEDs/USFEDs and DM appears to impair the metabolism [12], hampering any effort to lose weight while further complicating DM pathology. As per García-Mayor [12], the number of studies assessing metabolic control in patients with DM and EDs is still limited, with much of the published literature indicating either a lack of association, or the existence of a moderate association between the underlying ED and HbA_1c_ levels. On the other hand, some studies have reported worse metabolic outcomes among patients with T2DM and EDs [24,54]. Concerning ON, although the studies included herein indicate a trend towards poorer metabolic control, the reported differences were not significant.

According to Depa [55], achieving health and body weight control may be the main motives behind healthy eating among patients with DM and ON tendencies, although the extreme fear of gaining weight and body size overestimation—which are common in BN and AN patients—are typically lacking in ON patients [56]. ON is problematic, but is also seen as a “salvation” from chronic diseases. Women with DM in particular tend to be more preoccupied with their diet and body weight, demonstrating a greater frequency and severity of EDs [13,57]. Weight status appears to be a strong predictor of EDs, especially among women with overweight/obesity attempting to lose weight [10,58]. Given that most individuals with T2DM demonstrate excessive body weight, it becomes clear how this can easily propel disordered eating behavior. This is particularly important from a clinical perspective, as normal body weight or overweight can often mask EDs when clinicians are not cognizant [59], leaving the patients underrecognized and undertreated.

Meta-analyses of typical EDs in patients with DM offer an insight into the prevalence of a dual diagnosis in each age group and sex [60,61]; however, meta-analyses of ON do not appear to be feasible yet due to the lack of a consensus regarding the diagnostic criteria. Nevertheless, the recent call for a consensus on the diagnosis and signs associated with ON is bound to solve this issue [42].

Sex differences are often observed in the prevalence of USFED, although they are not always apparent, with the majority of studies indicating a greater prevalence among women. The effect of sex on the development of EDs/USFED among patients with DM appears to be highly influenced by age. Concerning ON, the results appear ambiguous, with some studies suggesting a higher prevalence among men. Many studies, however, indicate an increased prevalence of EDs in young patients with DM compared to their healthy peers, with these tendencies being more profound in females [13,62]. Young women with T1DM appear to be more prone to EDs and USFED and report engaging in disordered eating [15,63,64] more frequently than their male counterparts. Moreover, girls with T1DM also exhibit greater ED tendencies compared with girls of the same age without DM [62].

A characteristic of ON is a gradual intensification of dietary restrictions, often paired with obsessive thoughts and deviations from the imposed norms, leading to intense feelings of guilt, fear, shame, and additional dietary restrictions [41,65]. In the studies included herein, ON tendencies were associated with an increased fiber intake and a lower energy consumption. Restrictive diets often lead to an inadequate intake of micronutrients and a variety of diet-related complications, including osteopenia, anemia, hyponatremia, recurrent hypoglycemic episodes, and metabolic acidosis [4,66,67]. Other, less typical signs include a tendency to vegetarianism, a preoccupation with appearance, and calculating energy intake, often paired with food weighing [53]. Such outcomes and signs, however, were not recorded in the included studies.

According to Mitrofanova [68], diet-wise individuals with ON tendencies often fail to meet the dietary guidelines for most micronutrients, similarly to AN [69]. Research has also associated ON with an increased use of oral nutrient supplements (ONS) [70,71] in pursuit of improved health. However, the only study recording ONS intake herein failed to relate increased ON tendencies with supplementation in adults with T2DM [38].

According to Larrañaga [72], the risk of disordered eating is greater in patients with T1DM compared to the general population due to multiple interacting factors related to DM and its treatment [19,20]. On the other hand, a recent meta-analysis [73] revealed that EDs are highly prevalent in T2DM, as both BED and BN appeared to increase the risk of T2DM. Nevertheless, as the results are based on cross-sectional studies, it is difficult to discern whether EDs propel the development of overweight and T2DM, or the opposite. Concerning ON, none of the included studies compared patients having the two DM types (T1DM and T2DM) regarding the prevalence of ON tendencies.

### 4.2. Implications for Clinical Practice

According to Diabetes UK [74], the 7As model (aware, ask, assess, advise, assist, assign, and arrange) can be applied in clinical practice to identify patients with diabetes distress and hence likely to demonstrate OSFED or USFED. On the other hand, ON-specific treatment recommendations are lacking at the moment. In the case of a dual diagnosis (ED and DM), the Norwegian Knowledge Centre for the Health Services (NOKC) [75] suggests that treatment should follow a structured model that focuses on blood glucose control, the consumption of regular meals, and psychological treatment. Weekly group sessions are suggested for at least three months, although the quality of evidence is low and details on the correction of ON behaviors are not presented [75]. However, according to Zickgraf, the lack of evidence-based treatments for ON is apparent [76].

### 4.3. To Diagnose or Not?

At the moment, ON does not constitute an official psychiatric diagnosis, nor is it mentioned in the DSM-5 as a distinct ED [1,77]. It fits into the USFED domain (international classification of diseases ICD-10: 307.50, F50.9) among the diagnostic criteria that are still currently being discussed by experts using the Delphi method [42]. This, however, does not mean that any research conducted on this issue is in vain. As with every newly identified disorder, research is indispensable in aiding the development of a comprehensive definition and diagnostic criteria. Similarly, research on BED has long preceded the inclusion of the disorder in the DSM as a distinct entity. Moreover, although in the latest version of the DSM [1] the diagnostic criteria for AN were altered, that does not undermine the disorder as an entity or halt any relevant research; this indicates that even after an official psychiatric diagnosis is established, changes may still occur in the diagnostic criteria based on new evidence. Nevertheless, researchers are exhibiting a keen interest in ON research, with a growing amount of studies being produced in the past few years [77]. Furthermore, recent research conducted in the Netherlands [78] suggests that the majority of health professionals (78%) believe that ON should have its own diagnosis and position in the DSM, indicating that they acknowledge the problem and consider it to be separate from other EDs.

### 4.4. Limitations of the Study

The limitations of the present systematic review stem mainly from the small number of studies assessing ON tendencies among patients with DM. Moreover, due to the lack of a consensus regarding the diagnostic criteria, it was not feasible to perform a quantitative synthesis of the available evidence. Furthermore, due to the nature of the included studies (all were observational), we cannot surmise the prognosis of a dual diagnosis or its possible therapy.

## 5. Conclusions

In conclusion, the present systematic review of the literature points towards the fact that patients with DM may exhibit ON tendencies, although the exact prevalence cannot be calculated. Moreover, the number of primary research studies is still very limited with respect to defining the characteristics of persons with a dual DM–ON diagnosis or understanding the etiology and mechanistic drivers behind the development of ON. The clinicians and health professionals employed in DM treatment should be aware of the problem and assess ON tendencies in individuals who might present signs of an exaggerated, unhealthy obsession with healthy eating.

## Figures and Tables

**Table 1:** The PIO components of the study’s research question.

Population	Patients with prediabetes or diabetes mellitus (T1DM/T2DM)
Issue	Orthorexia nervosa
Outcomes	Symptoms, glycemic control.

T1DM, type 1 diabetes mellitus; T2DM, type 2 diabetes mellitus.

**Table 2:** The components of the SPIDER framework used for the identification of relevant studies.

Sample	Patients with Prediabetes or DM (T1DM/T2DM) of Any Age
Phenomenon of Interest	ON measures, tendencies, prevalence and correlates
Design	Original published research (any design, with emphasis on cross-sectional studies), including grey literature
Evaluation	Characteristics, views, experiences, prevalence
Research type	Quantitative and mixed methods peer-reviewed studies; grey literature including third-sector and government reports and briefings, educational theses, conference proceedings

ON, Orthorexia Nervosa; DM, Diabetes Mellitus; T1DM, Type 1 Diabetes Mellitus; T2DM, Type 2 Diabetes Mellitus.

## Data Availability

All data are presented within the manuscript text.
